# Neural signaling mechanisms in depression: bridging classical monoamine hypotheses, animal models, and emerging antidepressant strategies

**DOI:** 10.3389/fcell.2026.1777975

**Published:** 2026-03-25

**Authors:** Mizuki Yamamoto, Haruka Hirakata, Koji Toda

**Affiliations:** Department of Psychology, Keio University, Tokyo, Japan

**Keywords:** antidepressant mechanism, inflammation, ketamine, major depressive disorder, monoaminergic neurotransmission, psychedelics, rapid-acting antidepressants, stress

## Abstract

Major depressive disorder is a highly prevalent psychiatric condition that can affect individuals across the lifespan, yet its pathophysiology remains incompletely understood. Classical hypotheses, informed by preclinical and clinical studies, emphasized dysregulated monoaminergic neurotransmission and guided the development of widely prescribed antidepressants, including selective serotonin reuptake inhibitors and serotonin–noradrenaline reuptake inhibitors. Although these agents improved treatment outcomes, they typically require weeks to achieve therapeutic effects, must be taken continuously, and fail to produce adequate responses in nearly one-third of patients. In addition, adverse effects, such as increased suicidal ideation in some populations, highlight the need for safer and more effective therapies. Recent discoveries of rapid-acting antidepressant effects of ketamine and psychedelic compounds have challenged traditional monoaminergic models and highlighted alternative mechanisms involving glutamatergic signaling, synaptic plasticity, and immune–brain interactions. At the same time, long-standing assumptions about neurotransmitter abnormalities are being re-examined, reinvigorating interest in mechanistic and circuit-level models. This review summarizes historical and emerging perspectives on antidepressant development, outlines major animal models of depression, and highlights recent advances in translational research that are redefining therapeutic strategies.

## Highlights

- Classical monoaminergic theories guided early antidepressant development but remain insufficient to explain depression.
- Current antidepressants show delayed efficacy, require continuous use, and fail in approximately 30% of patients.
- Rapid-acting antidepressant effects of ketamine and psychedelics reveal non-monoaminergic mechanisms.
- Emerging evidence highlights roles for glutamatergic signaling, synaptic plasticity, and neuroimmune interactions.
- Stress-based and biologically driven animal models provide complementary insights into depressive pathology.
- Integrative mechanistic and circuit-level frameworks are reshaping future therapeutic strategies for major depressive disorders.

## 1 Introduction

Depression is a highly prevalent psychiatric disorder, affecting approximately 3.8% of the global population and representing a major global health burden. The disorder exhibits a pronounced sex difference, disproportionately affecting women, with an estimated 10% experiencing depressive symptoms during the perinatal period (World Health Organization, 2022). Despite its high prevalence and clinical impact, the pathophysiology of depression remains incompletely understood, reflecting the complex interplay of genetic, environmental, neurobiological, and immunological factors that contribute to disease onset and progression.

For several decades, disturbances in monoaminergic neurotransmission—particularly serotonergic signaling—have constituted a central framework for understanding depression and guiding pharmacological treatment. Accordingly, selective serotonin reuptake inhibitors (SSRIs) and serotonin–noradrenaline reuptake inhibitors (SNRIs) remain in first-line therapies. However, these treatments typically require weeks to achieve clinical efficacy, must be administered chronically, and fail to provide adequate relief for approximately one-third of patients with treatment-resistant depression (MSD Manuals, 2023). These clinical limitations suggest that a simplistic view of serotonin signaling as a uniform and global modulator of mood is insufficient to explain either disease mechanisms or therapeutic responses.

The emergence of rapid-acting antidepressants, most notably the N-methyl-D-aspartate (NMDA) receptor antagonist ketamine, has further challenged traditional monoamine-based models. Ketamine produces robust antidepressant effects within hours, yet its long-term safety and abuse potential remain concerns (Matveychuk et al., 2020). More recently, classic psychedelics such as psilocybin and lysergic acid diethylamide (LSD) have demonstrated rapid and sustained antidepressant effects in clinical studies, although their hallucinogenic properties pose ethical, safety, and regulatory challenges (McClure-Begley and Roth, 2022). Importantly, the efficacy of these pharmacologically diverse compounds suggests that antidepressant responses cannot be fully accounted for by changes in monoamine levels alone but instead may depend on circuit-level and functional reorganization within specific brain networks.

Within this broader re-evaluation of antidepressant mechanisms, increasing attention has been directed toward circuit-level models of mood regulation. In particular, the raphe–habenula circuitry—encompassing the dorsal raphe nucleus, median raphe nucleus, and their interactions with the lateral habenula—has emerged as a key network linking monoaminergic signaling with stress, aversion, and motivational control (Cameron et al., 2024; Hu et al., 2020; Nakamura, 2013; Ohmura and Nagayasu, 2025). The dorsal raphe nucleus and median raphe nucleus constitute major sources of forebrain serotonergic projections and exhibit pronounced cellular, molecular, and functional heterogeneity (Ogawa et al., 2014; Ohmura and Nagayasu, 2025). Accumulating evidence indicates that distinct subpopulations within these nuclei, together with their projection-specific connections to and from the lateral habenula, differentially regulate affective, motivational, and stress-related behaviors (Hu et al., 2020; Takahashi et al., 2022). Dysregulation of these interconnected circuits has been implicated in depressive-like phenotypes, suggesting that antidepressant efficacy may depend on selective modulation of specific subcircuits rather than global serotonergic tone.

To investigate such circuit-level mechanisms, well-validated animal models remain indispensable. Classical and contemporary behavioral paradigms, including the learned helplessness model, have provided critical insights into stress-induced behavioral adaptations and their underlying neural substrates. When combined with modern circuit-mapping, genetic, and pharmacological approaches, together with deep learning–based behavioral phenotyping and quantitative analysis, these models offer a powerful translational framework for linking molecular and cellular alterations to circuit dysfunction and behavioral outcomes relevant to depression.

In this review, we summarize historical and emerging perspectives on antidepressant development and outline major animal models used to study depression-related behaviors. Within this broader framework, we highlight recent advances in translational research, with particular emphasis on circuit-based and functional organization of the raphe–habenula network, to illustrate how evolving neurobiological concepts are reshaping therapeutic strategies for depression.

## 2 What is depression?

Depression is classified as a mood disorder and is characterized by a constellation of symptoms collectively referred to as a “major depressive episode.” The Diagnostic and Statistical Manual of Mental Disorders, Fifth Edition (DSM-5), published by the American Psychiatric Association, provides the most widely used diagnostic criteria in clinical and research settings (American Psychiatric Association, 2013). According to the DSM-5, individuals who experience depressive episodes without a history of mania are diagnosed with major depressive disorder, whereas those who exhibit both depressive and manic episodes are diagnosed with bipolar disorder.

A diagnosis of major depressive disorder requires the presence of at least one of two core symptoms—depressed mood and loss of interest or pleasure (anhedonia), the latter reflecting a reduced capacity to experience reward or positive affect—along with at least five additional symptoms persisting for a minimum of 2 weeks. These include: 1) decreased or increased appetite and significant weight loss or gain; 2) insomnia or hypersomnia; 3) psychomotor agitation or retardation; 4) fatigue or loss of energy; 5) feelings of worthlessness or excessive or inappropriate guilt; 6) diminished ability to think, concentrate, or make decisions; and 7) recurrent thoughts of death, suicidal ideation, or suicide attempts. The symptoms must occur nearly every day, cause significant distress or impairment in social, occupational, or other important areas of functioning, and cannot be better explained by a medical condition or substance use.

In addition to psychological symptoms, depression is frequently accompanied by somatic complaints such as headaches, muscle tension, pain syndromes, appetite changes, and gastrointestinal disturbances, which often co-occur with depressive states. Numerous psychosocial factors, including stressful life events, interpersonal difficulties, and major environmental changes (e.g., relocation, employment transitions, childbirth), can precipitate the onset of depressive symptoms. Although environmental stressors play a major role, intrinsic biological factors also contribute significantly to vulnerability. These include: 1) genetic predisposition influencing stress responsibility and neural plasticity; 2) neurological diseases such as cerebrovascular disorders; and 3) abrupt hormonal fluctuations associated with menstruation or childbirth. These biological factors are thought to modulate neurotransmitter systems and increase susceptibility to depression.

The DSM-5 reflects prevailing clinical and research frameworks and has facilitated advances in psychiatric classification and research (Regier et al., 2013). Nevertheless, concerns have been raised regarding potential overdiagnosis due to relatively broad diagnostic thresholds (Young, 2016). As a result, there is growing interest in identifying objective biomarkers that could improve diagnostic precision. Although no biomarker has yet been validated for clinical use, accumulating evidence discussed in the DSM-5 and related literature points to potential associations with hypothalamic–pituitary–adrenal (HPA) axis dysregulation, neurotrophic factors, and inflammatory cytokines (American Psychiatric Association, 2013).

Recent advances in basic neuroscience have accelerated our understanding of the physiological underpinnings of depression. In particular, substantial progress has been made in elucidating biological indicators such as neurotrophic signaling and inflammatory mediators, leading to active efforts to develop novel antidepressants that target these pathways. In this review, we summarize current perspectives on the pathophysiology of depression, existing and emerging antidepressant treatments, and major animal models used to investigate depressive-like states.

## 3 Pathophysiological hypotheses of depression

### 3.1 Monoamine hypothesis

One of the earliest and most influential biological frameworks proposed to explain the pathophysiology of depression is the monoamine hypothesis. As described by Cosci and Chouinard (2019), this hypothesis originated in the 1950s following observations that administration of reserpine induced depressive-like symptoms in patients. Reserpine depletes monoamines—particularly noradrenaline—by inhibiting their uptake into synaptic vesicles.

Subsequent findings that monoamine oxidase inhibitors (MAOIs) and tricyclic antidepressants (TCAs), such as imipramine, alleviate depressive symptoms further supported the notion that monoamine deficiency contributes to the development of depression. In its widely accepted contemporary form, the monoamine hypothesis posits that impaired signaling of serotonin, noradrenaline, and dopamine underlies depressive symptomatology.

### 3.2 Serotonin

Serotonin is synthesized from the essential amino acid tryptophan and functions as a peripheral hormone and a central neurotransmitter. In the central nervous system, serotonin is produced predominantly in the raphe nuclei—representing only 1%–2% of total body serotonin—and extensively projects to multiple brain regions where it regulates diverse functions including emotion, aggression, sleep–wake cycles, appetite, reward processing, and responses to aversive stimuli (Jacobs and Azmitia, 1992; Berger et al., 2009). The clinical efficacy of SSRIs, which increase synaptic serotonin availability and enhance serotonergic neurotransmission, has long been regarded as strong evidence supporting serotonergic involvement in depression (Cipriani et al., 2018).

However, the serotonin deficiency hypothesis has been increasingly challenged. An umbrella review by Moncrieff et al. (2023) concluded that depression is not consistently associated with reduced serotonin levels or activity, and that acute tryptophan depletion does not reliably induce depressive symptoms. These conclusions remain controversial, as critics have emphasized methodological limitations, misinterpretation of the reviewed findings, and insufficient consideration of the complex physiological consequences of tryptophan depletion (Jauhar et al., 2023). Although numerous studies and meta-analyses have examined the relationship between serotonin dysregulation and depression, the field has yet to reach a definitive consensus.

Another frequently cited argument against the serotonin hypothesis concerns the delayed onset of antidepressant efficacy. Despite rapidly increasing serotonin levels in the synaptic cleft, SSRIs typically require several weeks to produce clinical improvement, and approximately 30% of patients do not adequately respond to treatment. While these observations appear inconsistent with a simple serotonin-deficiency model, several mechanistic explanations have been proposed. Chronic SSRI administration may produce heterogeneous changes in serotonin levels across brain regions (Fritze et al., 2017). Additionally, the presence of inhibitory somatodendritic 5-HT_1A_ autoreceptors in the raphe nuclei can attenuate serotonergic neuronal firing, potentially limiting the expected increase in synaptic serotonin during early SSRI treatment (Commons and Linnros, 2019).

Thus, the delayed antidepressant effects of SSRIs do not necessarily invalidate the involvement of serotonergic dysfunction in depression. Instead, the substantial proportion of patients who fail to respond to SSRIs suggests that serotonin abnormalities are not universal across all individuals with depression, and that additional neurotransmitter systems and neural circuits likely play critical roles in the disorder’s etiology (Fries et al., 2023).

### 3.3 Noradrenaline

With respect to depression, alterations in noradrenergic signaling have been reported in unmedicated patients. Using PET imaging, Moriguchi et al. (2016) demonstrated increased indices of noradrenaline transmission in the thalamus and related regions, suggesting region-specific dysregulation of noradrenergic tone in depressive states. In contrast, postmortem studies have reported elevated expression of *α*_2_-adrenergic receptors (Rivero et al., 2014) and enhanced G-protein coupling at these receptors (Valdizán et al., 2010). Given that *α*_2_-adrenergic receptors are G_i/o_-coupled and function as inhibitory autoreceptors on noradrenergic neurons, these changes are generally interpreted as compensatory adaptations that may act to restrain excessive noradrenaline release or reflect homeostatic responses to chronic alterations in noradrenergic activity. These findings suggest that depression is not characterized by a uniform increase or decrease in noradrenergic signaling, but rather by complex, region- and circuit-specific dysregulation accompanied by compensatory receptor-level adaptations.

### 3.4 Dopamine

Dopamine, another major monoaminergic neurotransmitter, is synthesized and released by dopaminergic neurons that are particularly abundant in the ventral midbrain. These neurons give rise to several distinct pathways that underlie voluntary movement, motivation, reinforcement learning, and other complex behaviors (Franco et al., 2021; Chinta and Andersen, 2005). In the context of depression, considerable attention has focused on the relationship between dopaminergic dysfunction and anhedonia—a core symptom of the disorder (Yadid and Friedman, 2008; Belujon and Grace, 2017). Patients with depression characterized by pronounced anhedonia exhibit reduced dopamine transporter (DAT) binding across the bilateral striatum, including the caudate and putamen (Sarchiapone et al., 2006). These findings suggest alterations in DAT density or dopamine availability in the synaptic cleft, supporting the hypothesis that deficits in dopaminergic reward processing contribute to depressive symptoms.

### 3.5 γ-aminobutyric acid (GABA)

Beyond monoamines, increasing evidence implicates other neurotransmitter systems—most notably γ-aminobutyric acid (GABA), the principal inhibitory transmitter, and glutamate, the major excitatory transmitter—in the pathophysiology of depression. GABAergic synapses constitute approximately one-third of all synapses in the central nervous system and play a pivotal role in constraining glutamatergic excitatory transmission. Accordingly, the balance between excitatory glutamatergic and inhibitory GABAergic signaling, commonly referred to as the excitatory–inhibitory (E/I) balance, has emerged as a fundamental regulatory principle underlying normal brain function as well as a wide range of neuropsychiatric disorders.

In depression, particular attention has been directed toward dysfunction of the GABAergic system and its close association with dysregulation of the HPA axis (Fogaça and Duman, 2019). In mammals, stress activates corticotropin-releasing hormone (CRH) neurons located in the paraventricular nucleus (PVN) of the hypothalamus, leading to increased secretion of circulating corticosteroids—a hallmark of HPA axis activation. The activity of PVN CRH neurons is tightly regulated by GABAergic inhibitory synaptic inputs. Consistent with this regulatory role, pharmacological blockade of GABA_B_ receptors within the PVN enhances stress-induced elevations in circulating corticosterone levels (de Souza and Franci, 2008). Genetic evidence further supports the contribution of GABAergic dysfunction: mice lacking or carrying mutations in the γ2 subunit of the GABA_A_ receptor exhibit heightened HPA axis activity (Shen et al., 2010) as well as pronounced anxiety- and depression-like behaviors (Smith and Rudolph, 2012). Clinically, reduced GABA concentrations have been reported across multiple brain regions in patients with major depressive disorder, reinforcing the notion that impaired GABAergic signaling contributes to disease pathophysiology (Fogaça and Duman, 2019).

These observations have stimulated growing interest in GABA receptors as therapeutic targets for antidepressant development. Among such approaches, neurosteroids—particularly allopregnanolone, a progesterone-derived neurosteroid—have attracted considerable attention. Allopregnanolone acts as a potent positive allosteric modulator of GABA_A_ receptors and differs pharmacologically from classical benzodiazepines. Whereas benzodiazepines primarily enhance phasic inhibition by acting on γ2 subunit–containing synaptic GABA_A_ receptors, neurosteroids preferentially modulate δ subunit–containing extrasynaptic GABA_A_ receptors, thereby strengthening tonic inhibition and regulating baseline neuronal excitability (Meyer et al., 2022). Through this mechanism, neurosteroid-based GABA_A_ receptor modulators are thought to restore E/I balance at the circuit level, suppress excessive network activity, and normalize emotional regulation.

In addition to their effects on inhibitory transmission, neurosteroids including allopregnanolone have been shown to modulate HPA axis activity and enhance the expression of brain-derived neurotrophic factor (BDNF), suggesting that downstream plasticity-related signaling pathways may contribute to their rapid and sustained antidepressant effects (Naert et al., 2007). Building on these mechanistic insights, the U.S. Food and Drug Administration (FDA) approved zuranolone—a synthetic allopregnanolone analogue—as the first oral treatment specifically indicated for postpartum depression in 2023, marking a major milestone in GABA_A_ receptor–based antidepressant therapeutics.

Nevertheless, the clinical application of GABA-enhancing agents warrants careful consideration, particularly during pregnancy and early development. The antiepileptic drug valproate, which increases GABA levels, has been associated with an elevated risk of autism spectrum disorders in offspring exposed *in utero* (Christensen et al., 2013; Veroniki et al., 2017). Moreover, both excessive and insufficient placental levels of allopregnanolone have been suggested to increase susceptibility to autism spectrum disorders (Vacher et al., 2025). These findings demonstrate the importance of a cautious, mechanistically informed approach to the development and clinical use of GABAergic antidepressants, especially in vulnerable populations.

### 3.6 Glutamate

Another neurotransmitter system implicated in the pathophysiology of depression is the glutamatergic system. Trullas and Skolnick (1990) demonstrated that several NMDA receptor antagonists exert antidepressant-like effects in rodents, suggesting that signaling pathways governed by specific subtypes of ionotropic glutamate receptors may contribute to psychiatric disorders. Supporting this notion, stress exposure increases glutamate release in the hippocampus of rats (Moghaddam et al., 1994), and elevated brain glutamate levels have been reported in patients with bipolar disorder and major depressive disorder (Hashimoto et al., 2007).

Glutamate, an amino acid and the principal excitatory neurotransmitter in the central nervous system, mediates rapid synaptic excitation and is essential for learning, memory, and numerous complex cognitive and behavioral processes (Stallard and Ahmad, 2024). Since excessive glutamatergic signaling can induce excitotoxicity, extracellular glutamate levels are tightly regulated within the synaptic cleft (Riedel et al., 2003). Nearly all neurons in the brain express glutamate, which acts through two major classes of receptors: (1) G protein–coupled metabotropic glutamate receptors (mGluRs) and (2) ionotropic receptors, including NMDA receptors, α-amino-3-hydroxy-5-methyl-4-isoxazolepropionic acid (AMPA) receptors, kainate receptors, and δ-type receptors. Several of these receptor subtypes are also expressed in glial cells, further contributing to glutamatergic regulation. These ionotropic receptors mediate fast excitatory synaptic transmission on a millisecond timescale. Metabotropic glutamate receptors are classified into eight subtypes that are further divided into three groups based on sequence homology, signal transduction pathways, and pharmacological properties. Group I receptors, consisting of mGluR1 and mGluR5, are predominantly localized postsynaptically, whereas Group II (mGluR2 and mGluR3) and Group III receptors (mGluR4, mGluR6, mGluR7, and mGluR8) are mainly expressed presynaptically (Acher, 2011; Mao et al., 2022). Pharmacological modulation of mGluRs is generally thought to regulate glutamatergic neurotransmission in a modulatory manner, without directly disrupting basal synaptic transmission. For this reason, these receptors have attracted considerable interest as potential therapeutic targets, particularly for the development of novel antidepressant strategies (Acher, 2011).

Among G protein–coupled receptors, antagonists and negative allosteric modulators of mGluR5—such as MPEP and its derivative MTEP—have attracted significant attention as potential antidepressant agents (Wierońska and Pilc, 2009). Previous studies show that mGluR5 inhibition produces anxiolytic-like effects (Brodkin et al., 2002), and chronic treatment with several antidepressants, including SSRIs, increases the binding of MPEP to mGluR5 in the hippocampus and prefrontal cortex (Nowak et al., 2014). In addition, postmortem studies have reported elevated levels of mGluR2/3 in the prefrontal cortex of patients with major depressive disorder (Feyissa et al., 2010), and numerous preclinical studies demonstrate context-dependent antidepressant-like effects of mGluR2/3 antagonists (Pilc et al., 2008).

Despite this accumulating evidence, many fundamental aspects of mGluR biology—and their precise roles in the development and treatment of psychiatric disorders—remain unresolved. Continued investigation is therefore essential for understanding how glutamatergic signaling contributes to depression and for identifying new therapeutic targets within this complex neurotransmitter system.

A major breakthrough in antidepressant research emerged with the discovery of ketamine’s rapid antidepressant effects. Ketamine acts primarily as a noncompetitive NMDA receptor antagonist, leading indirectly to enhanced AMPA receptor–mediated transmission. Its clinical efficacy drew significant attention to the link between depression and neuroplasticity. Nearly 3 decades ago, Duman et al. (1997) proposed the “neurotrophic hypothesis of depression,” which posits that impairments in neurotrophic signaling contribute to depressive symptomatology and that effective treatments may restore synaptic plasticity.

Monoaminergic, GABAergic, and glutamatergic systems should not be viewed as independent contributors to depression, but rather as interacting components of distributed neural circuits that regulate mood, motivation, and stress responsiveness. Monoamines such as serotonin, noradrenaline, and dopamine modulate the gain and plasticity of glutamatergic transmission within cortico–limbic networks, while GABAergic interneurons shape the temporal precision and stability of these circuits. Dysregulation at any of these levels can converge on maladaptive circuit states characterized by impaired reward processing, altered stress coping, and behavioral withdrawal. From this perspective, the therapeutic effects of both conventional antidepressants and rapid-acting agents such as ketamine and psilocybin may be understood as circuit-level recalibrations mediated through distinct but convergent molecular mechanisms.

### 3.7 Neurotrophic factors

Neurotrophic factors are proteins that regulate neuronal survival, development, and differentiation. Among them, BDNF is the most abundant and widely distributed neurotrophin in the central nervous system. BDNF binds to its high-affinity receptor, tropomyosin receptor kinase B (TrkB), which activates three major intracellular signaling pathways—mitogen-activated protein kinase (MAPK), phospholipase C-γ (PLC-γ), and phosphatidylinositol-3-kinase (PI3K). These pathways orchestrate neuronal development, synaptic maintenance, and cell survival across the lifespan (Arosio et al., 2021). Notably, activation of the BDNF–TrkB–PLC-γ cascade promotes presynaptic calcium release, increases the number of synaptic vesicles, and facilitates glutamate release (Fries et al., 2023), processes that are fundamental to synaptic strengthening and plasticity.

A substantial body of evidence suggests that BDNF signaling is disrupted in depression. Numerous animal models of depression demonstrate reduced BDNF expression in the hippocampus, and it has been proposed that current antidepressant treatments may exert their therapeutic effects, at least in part, by inducing structural and functional changes mediated through neurotrophic pathways (Duman and Monteggia, 2006). Consistent with this hypothesis, chronic administration of antidepressants or electroconvulsive therapy increases hippocampal BDNF mRNA levels in rodents (Nibuya et al., 1995), and clinical studies have shown that prolonged SSRI treatment elevates peripheral BDNF levels in patients (Ramesh et al., 2021). However, whether treatment-induced changes in BDNF are causally related to clinical improvement remains a matter of active debate (Madsen et al., 2024).

These findings highlight the central role of neuroplasticity in depression and emphasize the importance of glutamatergic and neurotrophic mechanisms—particularly those engaged by ketamine—in advancing novel therapeutic strategies.

### 3.8 Neuroendocrine system

Another major line of evidence implicates dysregulation of the neuroendocrine system—particularly the HPA axis—in the pathophysiology of depression. The HPA axis is a central component of the stress-response system and is initiated by neurons in the paraventricular nucleus (PVN) of the hypothalamus. PVN neurons synthesize and release CRH, which acts on the anterior pituitary to stimulate the secretion of adrenocorticotropic hormone (ACTH). ACTH subsequently drives the synthesis and release of glucocorticoids from the adrenal cortex. Glucocorticoids exert their effects primarily via two receptor types—glucocorticoid receptors and mineralocorticoid receptors. Glucocorticoid receptors are expressed in hippocampal CA1 and dentate gyrus regions, as well as in cortical and thalamic areas, whereas mineralocorticoid receptors are densely concentrated in the hippocampus (Mahfouz et al., 2016). These receptor systems enable the hippocampus to play a pivotal role in negative feedback regulation of the HPA axis.

Recent studies have begun to clarify the neural circuitry underlying this feedback control. Optogenetic manipulation of the ventral hippocampus revealed that GABAergic neurons in the bed nucleus of the stria terminalis inhibit excitatory hippocampal projections to the PVN, thereby suppressing HPA-axis activation (Cole et al., 2022). Under normal conditions, glucocorticoids facilitate adaptive responses to stress; however, insufficient or excessive HPA-axis activation can contribute to pathological states (Smith and Vale, 2006).

One influential account linking HPA-axis dysfunction to depression is the “glucocorticoid neurotoxicity hypothesis.” This hypothesis posits that prolonged exposure to elevated glucocorticoids renders neurons particularly vulnerable to damage, thereby increasing susceptibility to neurotoxic insults and accelerating neuronal attrition. Such processes are thought to contribute to structural changes, including hippocampal atrophy, observed in depression (Sheline, 2011). Excessive glucocorticoid signaling has been shown to reduce survival and impair maturation of proliferating progenitor cells in the dentate gyrus (Wong and Herbert, 2006). Meta-analytic evidence further supports reduced hippocampal volume in patients with major depressive disorder (Videbech and Ravnkilde, 2004). However, the causal direction of the relationship between hippocampal atrophy and depressive onset remains unresolved, and further investigation is needed to determine whether volumetric reductions are a cause, consequence, or epiphenomenon of depressive pathology (Sheline, 2011).

Importantly, HPA-axis dysregulation provides a mechanistic bridge between environmental stress exposure and neural circuit alterations implicated in depression. Excessive or prolonged glucocorticoid signaling can impair hippocampal and prefrontal cortical function, disrupt negative feedback regulation, and alter synaptic plasticity within stress- and reward-related circuits (Mizoguchi et al., 2003). These neuroendocrine effects interact closely with monoaminergic and glutamatergic signaling, suggesting that stress hormones do not act in isolation but modulate the same circuit-level substrates targeted by antidepressant interventions.

### 3.9 Immune system

In recent years, the relationship between the immune system and psychiatric disorders, including depression, has attracted increasing attention. The first systematic proposal of this connection was made by Smith (1991), who observed an elevated risk of depression in patients with rheumatoid arthritis and noted that administration of tumor necrosis factor-α (TNF-α) in patients undergoing cancer treatment could induce depressive symptoms. Based on these clinical observations, Smith proposed that cytokines released by macrophages may contribute to the development of depression—a concept now known as the “macrophage theory of depression.”

Smith (1991) also suggested that the higher incidence of depression in women aged 18–44 could be related to the pro-inflammatory effects of estrogen. However, contemporary epidemiological evidence indicates that depression can onset across multiple life stages, including adolescence, the peripartum period, and late adulthood, making it overly simplistic to attribute depressive vulnerability solely to estrogen-mediated inflammation. Similarly, Smith’s attempt to explain the lower prevalence of depression in Japan compared to the United States by the anti-inflammatory effects of eicosapentaenoic acid (EPA) from fish consumption is problematic, as the low rates of diagnosed depression and suicide in Japan are likely influenced by sociocultural factors, including underreporting of depressive symptoms (Mahlich et al., 2018). Nonetheless, EPA has been shown to exert anti-inflammatory effects *in vitro* and *in vivo* (Patted et al., 2024). While not all of Smith’s initial hypotheses remain fully supported, his early work was pioneering in highlighting the potential role of inflammation in depression at a time when the monoamine hypothesis dominated, and it laid the groundwork for subsequent investigations into immune and metabolic contributions to depressive pathophysiology.

Subsequent research over the past 2 decades has provided more quantitative evidence linking inflammation and depression. Numerous meta-analyses and clinical studies have consistently reported elevated levels of circulating pro-inflammatory cytokines, such as interleukin-6 (IL-6) and TNF-α, in patients with major depressive disorder (Dowlati et al., 2010; Lanquillon et al., 2000; Tuglu et al., 2003). Clinically, administration of interferon-α (IFN-α) to patients has been shown to induce depressive-like states, further supporting a causal link between peripheral inflammation and mood disturbances (Raison et al., 2006).

Mechanistically, Jin et al. (2024) demonstrated that peripheral cytokine concentrations are monitored by the vagus nerve, with signals transmitted to the caudal nucleus of the solitary tract, allowing the central nervous system to regulate systemic inflammatory responses. Specifically, calcitonin/calcitonin-related polypeptide alpha (CALCA)-expressing vagal neurons transmit pro-inflammatory signals, whereas TRPA1-expressing neurons convey anti-inflammatory signals to the caudal solitary nucleus, highlighting the vagus nerve’s critical role in integrating peripheral and central immune signaling.

These insights have also led to the development of neuromodulatory interventions for treatment-resistant depression. Vagus nerve stimulation was approved by the FDA in 2005 as a therapeutic option for refractory depression (Austelle et al., 2022). Early pilot studies reported reductions in depression scores following vagus nerve stimulation (Rush et al., 2000), and some long-term follow-up studies suggested sustained improvement over one to 2 years (Rush et al., 2000; Bajbouj et al., 2010). However, subsequent systematic reviews concluded that evidence from clinical trials remains limited, leaving efficacy uncertain (Lv et al., 2019). Recent clinical trials indicate that while standardized depression rating scales may not show significant differences between intervention and control groups, clinician- and rater-assessed outcomes often reveal significant improvements with vagus nerve stimulation (Conway et al., 2025), suggesting that further investigation is warranted.

These findings support a model in which peripheral immune activation influences mood and behavior through defined neuroimmune communication pathways, including vagal afferent signaling and cytokine-mediated modulation of synaptic function. In this framework, inflammation contributes to depression not merely as a peripheral correlate, but as an active modulator of neural circuits governing motivation, reward, and stress sensitivity. Such immune–brain interactions may underlie a biologically distinct subtype of depression, with important implications for patient stratification and the development of targeted anti-inflammatory or neuromodulatory treatments.

## 4 Historical conceptualizations of depression

### 4.1 From humoral theory to moral treatment

From classical antiquity through the early modern period, depressive states were primarily conceptualized within prevailing philosophical and medical frameworks (Jackson, 1986). In Hippocratic medicine, melancholia was attributed to an excess of black bile within the humoral system, and treatment emphasized restoration of balance through diet, exercise, and regulation of daily life. During the Greco-Roman period, therapeutic strategies expanded to include lifestyle modification and various somatic interventions, while also acknowledging the role of emotional distress. By the 17th and 18th centuries, advances in anatomy and physiology, together with sensationalist philosophy, gradually shifted attention toward the brain and nervous system as substrates of mental function. This intellectual transition enabled early neuropsychological explanations of depression and laid the foundation for moral treatment, which emphasized structured social engagement, psychological guidance, and environmental modulation as therapeutic tools.

### 4.2 Emergence of diagnostic frameworks and psychological theories of depression

In the late 19th and early 20th centuries, psychiatry adopted more systematic diagnostic frameworks, most notably through Kraepelin’s formulation of manic-depressive illness, which contributed to the differentiation of mood disorders as distinct clinical entities (Porter, 2002; Shorter, 1997). In parallel, psychological theories of depression emerged, culminating in psychoanalytic models that emphasized intrapsychic conflict and early life experiences. Although classical psychoanalysis showed limited efficacy for depression, it exerted a lasting influence on later psychotherapeutic approaches, including cognitive therapy and cognitive-behavioral therapy, which remain central evidence-based treatments.

### 4.3 Early somatic and shock-based treatments for depression

Concurrently, interest in somatic interventions increased, motivated in part by observations that intense physiological stimulation could transiently alleviate depressive symptoms. Early 20th-century biological approaches included fever therapy, prolonged sleep therapy (Windholz et al., 1993), and various forms of shock treatment such as insulin coma therapy (Sakel, 1956; Shorter, 1997) and chemically or electrically induced convulsions (Gazdag et al., 2009). Among these, electroconvulsive therapy, introduced in 1938, demonstrated robust and enduring efficacy for severe depression. Although many early somatic treatments were later abandoned due to safety and ethical concerns, they reinforced the notion that direct modulation of brain function could profoundly influence mood states.

### 4.4 The emergence of modern psychopharmacology

A major turning point in psychopharmacology occurred in 1949 with Cade’s demonstration of lithium’s efficacy in mania, paving the way for modern biological treatments of mood disorders (Cade, 1949; Healy, 1999). Subsequent decades witnessed the introduction of antidepressant and antipsychotic medications, including imipramine and chlorpromazine, which catalyzed the development of neurochemical theories of depression and established pharmacotherapy as a central component of clinical management.

## 5 Antidepressants

### 5.1 Monoamine oxidase inhibitors (MAOIs)

The modern era of antidepressant pharmacotherapy began with the near-simultaneous serendipitous discovery of two compounds with mood-elevating properties: MAOIs and the tricyclic antidepressant imipramine (Healy, 2000). Initial clinical observations in the early 1950s revealed that iproniazid, an anti-tuberculosis drug, produced marked improvements in energy, appetite, sleep, and social engagement in patients, along with occasional psychomotor excitation (López-Muñoz and Alamo, 2009; Sandler, 1990; Selikoff et al., 1952). In animal models, Chessin et al. (1957) demonstrated that iproniazid attenuated reserpine-induced depressive-like states, supporting its potential antidepressant effects.

MAOIs exert their pharmacological actions by inhibiting the enzymatic breakdown of monoamines—including serotonin, noradrenaline, and dopamine—thereby increasing their availability in the synaptic cleft. Following their introduction, widespread clinical use was tempered by significant adverse effects, such as hypertensive crises related to dietary tyramine and drug interactions, leading to a decline in MAOI prescriptions with the advent of safer agents and the introduction of TCAs (Kline and Cooper, 1980).

### 5.2 Tricyclic antidepressants (TCAs)

The discovery of imipramine emerged from early research into antipsychotic and antihistaminic compounds. Chlorpromazine, developed from promethazine, exhibited central nervous system effects that stimulated interest in related structures (Shen, 1999). Roland Kuhn and colleagues at Geigy subsequently identified imipramine in the 1950s as having significant antidepressant effects in clinical observations (Cahn, 2006).

TCAs are defined by their three-ring core structure and exert antidepressant effects primarily through inhibition of monoamine reuptake, increasing synaptic concentrations of noradrenaline and/or serotonin. Although TCAs, including amoxapine and nortriptyline, were widely prescribed due to efficacy, their use has been limited by adverse effects associated with anticholinergic, antihistaminic, and antiadrenergic activities (Glassman and Bigger, 1981; Kamp et al., 2024). In response, tetracyclic antidepressants were developed with improved tolerability, though they generally display weaker antidepressant potency (Richelson, 2001; Stahl, 2021).

### 5.3 Selective serotonin reuptake inhibitors (SSRIs)

Although the molecular structures of early antidepressants were broadly similar, Paul Kielholz, then a professor at the University of Basel, noted that individual TCAs appeared to differ in their clinical profiles. In “Diagnose und Therapie der Depressionen für den Praktiker”, Kielholz (1968) reported that imipramine and clomipramine were particularly effective for depressed mood and sadness, protriptyline and nortriptyline for apathy and reduced motivation, and amitriptyline and doxepin for anxiety and agitation. While these observations were primarily based on clinical experience rather than controlled trials, they suggested that TCAs might exert partially distinct therapeutic effects.

These clinical impressions attracted the attention of Arvid Carlsson, who later received the Nobel Prize in Physiology or Medicine for his contributions to elucidating monoaminergic neurotransmission. Carlsson et al. (1969) systematically examined the inhibitory effects of various TCAs on serotonin and noradrenaline reuptake in rodents, demonstrating substantial variability in their relative potencies for inhibiting these monoamines. Integrating these pharmacological findings with Kielholz’s clinical observations, Carlsson proposed that serotonin reuptake inhibition was more closely associated with improvements in mood, whereas noradrenaline reuptake inhibition may preferentially influence motivation and psychomotor activity.

Based on this conceptual framework, Carlsson further hypothesized that selective enhancement of serotonergic neurotransmission might represent an effective and potentially safer therapeutic strategy for depression, thereby contributing to the rationale for the development of SSRIs (Carlsson, 2018). While working at Astra, Carlsson and colleagues modified the molecular structure of the antihistamine chlorpheniramine, ultimately leading to the development of zimelidine. In 1972, zimelidine became the first SSRI approved for clinical use and was marketed in Europe under the name Zelmid. Despite its initial promise, zimelidine was subsequently withdrawn due to its association with Guillain–Barré syndrome. In parallel, Bryan B. Molloy at Eli Lilly independently developed fluoxetine, which was introduced to the market in 1974 under the name Prozac (Healy, 2000). Fluoxetine later became one of the most widely prescribed antidepressants worldwide.

Despite their ability to rapidly inhibit serotonin reuptake, selective serotonin reuptake inhibitors (SSRIs) typically require several weeks to produce clinically meaningful antidepressant effects. Moreover, particularly during the early phase of treatment, SSRI use has been associated with an increased risk of adverse effects, including suicidal ideation in children and adolescents (Stone et al., 2009). One proposed mechanism underlying the delayed onset of therapeutic efficacy involves 5-HT_1A_ autoreceptors expressed presynaptically in the raphe nuclei, which exert inhibitory control over serotonergic neuron firing. Acute increases in synaptic serotonin following SSRI administration can activate these autoreceptors, transiently suppressing neuronal firing and reducing serotonin release to projection areas. With chronic SSRI treatment, reduced 5-HT_1A_ receptor binding (Gray et al., 2013) and functional desensitization of presynaptic 5-HT_1A_ autoreceptors have been reported (Blier and de Montigny, 1994), changes that are thought to permit a gradual enhancement of serotonergic transmission.

Importantly, however, 5-HT_1A_ receptors are not restricted to presynaptic autoreceptors, but are also widely expressed postsynaptically in cortical and limbic regions, including the hippocampus and prefrontal cortex, where they modulate neuronal excitability and emotional behavior (Richardson-Jones et al., 2010; Richardson-Jones et al., 2011). Genetic and pharmacological studies have demonstrated that presynaptic and postsynaptic 5-HT_1A_ receptor populations play distinct and sometimes opposing roles in the regulation of stress responses and antidepressant-like behaviors. Furthermore, because desensitization of presynaptic 5-HT_1A_ autoreceptors alone does not fully normalize serotonergic firing rates, the delayed therapeutic effects of SSRIs are likely to involve slower neuroplastic adaptations, including postsynaptic receptor signaling, synaptic remodeling, and circuit-level changes that extend beyond autoreceptor mechanisms (Commons and Linnros, 2019).

Consistent with this complexity, activation of the median raphe nucleus has been shown to induce aversive responses, reduce reward sensitivity (Kawai et al., 2022), and elicit anxiety-like behaviors (Abela et al., 2020; Ohmura et al., 2014; Ohmura et al., 2020). These findings raise the possibility that serotonergic signaling originating from the median raphe nucleus may contribute to some of the adverse effects observed during the initial stages of SSRI treatment. Furthermore, approximately 30% of patients fail to show a robust antidepressant response even after prolonged SSRI administration, suggesting the likelihood that the pathophysiology of depression cannot be fully explained by monoamine deficiency alone, including alterations in serotonergic signaling (Fries et al., 2023).

### 5.4 Serotonin–noradrenaline reuptake inhibitors (SNRIs)

SNRIs are a class of antidepressant agents that increase synaptic levels of serotonin and noradrenaline by inhibiting the serotonin transporter (SERT) and the noradrenaline transporter (NET) on presynaptic terminals. In contrast to SSRIs, which primarily enhance serotonergic neurotransmission, SNRIs act on both serotonergic and noradrenergic systems. This dual mechanism has been proposed to contribute to their therapeutic effects in depressive disorders.

Multiple SNRIs have been developed to date, and these compounds differ in their relative affinity and inhibitory potency toward SERT and NET. For instance, venlafaxine exhibits a dose-dependent pharmacological profile, with predominant inhibition of SERT at lower doses and increasing NET inhibition at higher doses (Aldosary et al., 2022). By comparison, duloxetine and milnacipran display more balanced inhibitory effects on both SERT and NET across clinically relevant dose ranges (Bymaster et al., 2005; Vaishnavi et al., 2004).

In clinical settings, SNRIs have generally been reported to demonstrate antidepressant efficacy comparable to that of SSRIs in the treatment of major depressive disorder, although some studies have suggested potential advantages in specific patient subgroups (Machado and Einarson, 2010; Bauer et al., 2009; Rodoshi et al., 2025). Noradrenergic facilitation has been hypothesized to contribute to improvements in symptoms such as reduced motivation, fatigue, and psychomotor retardation; however, the extent to which these effects translate into consistent clinical benefits remains under investigation (Singh et al., 2013). In addition, duloxetine and milnacipran have been evaluated for their potential analgesic effects in conditions such as fibromyalgia and neuropathic pain, but the current clinical evidence is mixed and continues to be an area of active research (Birkinshaw et al., 2024; Cording et al., 2015).

Regarding tolerability, meta-analyses have indicated that SNRIs are associated with lower tolerability and higher discontinuation rates compared with SSRIs (Cipriani et al., 2018). Furthermore, similar to SSRIs, SNRIs typically require several weeks of treatment to achieve clinically meaningful antidepressant effects, and a substantial proportion of patients do not respond adequately. These factors represent important limitations of SNRIs in routine clinical practice and highlight the ongoing need for alternative therapeutic strategies.

### 5.5 Noradrenergic and specific serotonergic antidepressants (NaSSAs)

Mirtazapine, developed in the Netherlands in 1989, is an antidepressant agent with a pharmacological profile that differs from that of SSRIs and SNRIs (Hassanein et al., 2023). It is classified as a noradrenergic and specific serotonergic antidepressant (NaSSA). In contrast to antidepressants that primarily exert their effects through inhibition of monoamine reuptake, NaSSAs modulate both noradrenergic and serotonergic neurotransmission through receptor-level mechanisms.

Pharmacologically, mirtazapine antagonizes central *α*_2_-adrenergic receptors located both as autoreceptors on noradrenergic neurons and as heteroreceptors on non-noradrenergic terminals. Blockade of presynaptic *α*_2_ autoreceptors relieves negative feedback inhibition of noradrenaline release, thereby enhancing noradrenergic neurotransmission. This increased noradrenergic activity, together with antagonism of *α*_2_ heteroreceptors on monoaminergic neurons, contributes to enhanced serotonergic neurotransmission. In addition, mirtazapine blocks postsynaptic 5-HT_2A_ and 5-HT_3_ receptors, biasing serotonergic signaling toward 5-HT_1A_ receptor–mediated pathways (Anttila and Leinonen, 2001). Through this combination of actions, mirtazapine modulates monoaminergic neurotransmission via mechanisms distinct from classical reuptake inhibition.

Clinical studies have reported that mirtazapine exhibits antidepressant efficacy broadly comparable to that of SSRIs and TCAs, while differing in its side-effect profile (Scott, 1999). In particular, a lower incidence of activating adverse effects, insomnia, sexual dysfunction, and gastrointestinal symptoms has been noted in some studies; however, tolerability profiles may vary across patient populations and treatment settings. As mirtazapine ultimately modulates noradrenergic and serotonergic systems, its antidepressant effects are generally considered to remain within the conceptual framework of the monoamine hypothesis of depression.

Since the introduction of SSRIs, additional antidepressant classes, including SNRIs and NaSSAs, have expanded the available pharmacological options. Nevertheless, these agents have not fully addressed key limitations of conventional antidepressant therapies, such as delayed onset of action and incomplete treatment response. Against this background, ketamine has emerged as a mechanistically distinct agent, prompting renewed interest in non-monoaminergic approaches to antidepressant development.

### 5.6 Ketamine

Ketamine, originally designated CI-581, is a phencyclidine (PCP) derivative synthesized by Calvin Stevens in 1962 and was initially developed for use as an anesthetic (Li and Vlisides, 2016). Owing to its rapid onset of action and favorable anesthetic profile, ketamine was widely adopted in clinical anesthesia. However, its clinical use was later restricted because emergence phenomena, including hallucinations, delusions, and confusion, were observed in some patients. In addition, recreational misuse of ketamine became a public health concern in North America during the 1970s and 1980s (Savić Vujović et al., 2023).

The potential antidepressant properties of ketamine were first reported in a clinical study published in 2000. Berman et al. (2000) demonstrated that sub-anesthetic doses of intravenous ketamine were associated with rapid reductions in depressive symptoms. Subsequent clinical studies reported that a single infusion could be associated with symptom improvement lasting several days to approximately 1 week, while repeated administration protocols were linked to sustained symptom reduction in patients with treatment-resistant depression (Phillips et al., 2019). Meta-analyses further support the presence of rapid antidepressant effects within 24 h following ketamine administration, with effects persisting for up to 7 days after a single dose and for several weeks following repeated dosing regimens (Kryst et al., 2020).

Ketamine’s pharmacological profile differs from that of conventional antidepressants and is primarily characterized by non-competitive antagonism of the NMDA receptor. Preclinical studies have implicated multiple molecular pathways in its antidepressant-like effects, including modulation of NMDA and AMPA receptor signaling, inhibition of glycogen synthase kinase-3β (GSK-3β), and engagement of BDNF–TrkB and mammalian target of rapamycin (mTOR) signaling pathways.

GSK-3β is a serine/threonine protein kinase involved in diverse cellular processes, including energy metabolism, synaptic plasticity, gene expression, and apoptosis. Experimental evidence suggests that ketamine may influence synaptogenic processes through inhibition of GSK-3β (Liu et al., 2013), and genetic studies have shown reduced antidepressant-like responses to ketamine in mice lacking GSK-3β inhibition (Beurel et al., 2011). However, pharmacological inhibition of GSK-3β alone does not fully reproduce ketamine’s behavioral effects in animal models of depression (Ma et al., 2013), indicating that GSK-3β inhibition represents one component of a broader and more complex molecular mechanism.

NMDA and AMPA receptors are ionotropic glutamate receptors that play central roles in excitatory neurotransmission. Ketamine acts as an open-channel blocker of NMDA receptors, and its inhibition of NMDA receptor-mediated signaling has been proposed to initiate downstream processes that enhance synaptic protein synthesis and plasticity (Autry et al., 2011; Miller et al., 2014). Importantly, ketamine-induced synaptic changes are considered mechanistically distinct from classical NMDA receptor–dependent long-term potentiation. Instead, transient suppression of NMDA receptor activity appears to trigger a rapid homeostatic response involving increased translation of proteins such as BDNF (Kavalali and Monteggia, 2025). This process is thought to engage mTOR signaling cascades and promote synaptogenesis and functional synaptic strengthening. Accumulating evidence also supports a critical contribution of AMPA receptor–mediated transmission to ketamine’s antidepressant-like effects. Several studies have shown that pharmacological blockade of AMPA receptors with 2,3-dihydroxy-6-nitro-7-sulfamoyl-benzo [f]quinoxaline-2,3-dione (NBQX) attenuates the behavioral and synaptic effects of ketamine, suggesting that enhanced AMPA receptor signaling is required for its full antidepressant-like profile (Koike and Chaki, 2014; Autry et al., 2011; Zanos et al., 2016; Zhou et al., 2014).

BDNF, a member of the neurotrophin family, is widely expressed in the central nervous system and has emerged as a key molecular mediator of the synaptic plasticity underlying ketamine’s antidepressant effects. Autry et al. (2011) demonstrated that the sustained inhibition of NMDA receptors at rest rapidly promotes BDNF translation via suppression of eukaryotic elongation factor 2 kinase (eEF2 kinase, also known as CaMKIII), thereby enhancing synaptic plasticity and contributing to ketamine’s long-lasting antidepressant effects. Furthermore, AMPA receptor activation in hippocampal slices has been shown to increase dendritic protein translation through BDNF release and TrkB receptor activation (Jourdi et al., 2009). Ketamine administration may also enhance mTOR signaling and BDNF levels in the hippocampus and prefrontal cortex via AMPA receptor-dependent mechanisms (Zhou et al., 2014). Supporting this, infusion of BDNF-neutralizing antibodies into the medial prefrontal cortex has been reported to abolish ketamine’s antidepressant effects in the forced swim test (Lepack et al., 2015).

### 5.7 Stereoisomers of ketamine

The identification of ketamine’s rapid antidepressant effects has provided important insights into the role of synaptic plasticity in mood disorders and treatment response (Krystal et al., 2024). Nonetheless, its clinical application remains constrained by regulatory status and adverse effects. These limitations have stimulated interest in ketamine stereoisomers, particularly (*R*)-ketamine and (*S*)-ketamine. While (*S*)-ketamine exhibits higher affinity for NMDA receptors, preclinical studies suggest that (*R*)-ketamine may produce more sustained antidepressant-like effects with fewer behavioral side effects (Zanos et al., 2018). Furthermore, evidence indicates that ketamine’s antidepressant actions may not depend exclusively on NMDA receptor antagonism, as (*R*)-ketamine produces antidepressant-like effects with minimal NMDA receptor inhibition (Zanos et al., 2016).

### 5.8 Metabolites of ketamine

Additional studies have proposed that ketamine metabolites, particularly (2*R*,6*R*)-hydroxynorketamine (HNK), may contribute to antidepressant-like effects. Ketamine undergoes hepatic metabolism via CYP2B6 and CYP3A4 to form norketamine, which is subsequently converted to hydroxynorketamine and dehydronorketamine. Among these metabolites, (2*R*,6*R*)-HNK and (2*S*,6*S*)-HNK are predominant in plasma and brain tissue following ketamine administration (Zanos and Gould, 2018). In rodent models, (2*R*,6*R*)-HNK has been shown to elicit antidepressant-like behavioral effects (Pham et al., 2018). Lumsden et al. (2019) systematically evaluated the behavioral and pharmacological effects of (2*R*,6*R*)-HNK and demonstrated that doses producing antidepressant-like effects did not correspond to levels required for substantial NMDA receptor antagonism. These findings support the possibility that (2*R*,6*R*)-HNK exerts its effects through mechanisms distinct from direct NMDA receptor blockade. Early clinical investigations of (2*R*,6*R*)-HNK are currently underway. A phase 1 study reported that single doses ranging from 0.1 to 4.0 mg/kg were not associated with sedation or dissociative symptoms (Raja et al., 2024), and a phase 2 clinical trial is ongoing to assess its efficacy in patients with depression (CenterWatch, 2025). These findings highlight the translational potential of ketamine and its metabolites while emphasizing the need for continued systematic evaluation in both preclinical and clinical settings.

In addition to (2*R*,6*R*)-HNK, the enantiomer (2*S*,6*S*)-HNK has also been reported to produce antidepressant-like effects in preclinical studies. For example, (2*S*,6*S*)-HNK elicited both acute and sustained antidepressant-like behavioral responses in stressed mouse models, including repeated social stress paradigms, without ketamine-related side effects (Kawatake-Kuno et al., 2024). Similarly, in a chronic corticosterone-induced depression model, (2*S*,6*S*)-HNK reduced measures of behavioral despair and anhedonia at acute and later time points (Yokoyama et al., 2020). These findings suggest that multiple ketamine metabolites may contribute to antidepressant actions through mechanisms that are partially distinct from NMDA receptor inhibition.

### 5.9 Group II metabotropic glutamate receptors

With the emergence of antidepressant strategies targeting glutamatergic neurotransmission, including ketamine, increasing attention has been directed toward antagonists and negative allosteric modulators of group II metabotropic glutamate receptors (mGluR2/3) as potential antidepressant approaches (Acher, 2011). mGluR2/3 are predominantly localized at presynaptic sites, where they regulate glutamate release, and pharmacological inhibition of these receptors has been shown to enhance glutamatergic transmission. Preclinical evidence indicates that, similar to ketamine, the antidepressant-like effects of mGluR2/3 antagonists critically depend on AMPA receptor–mediated signaling (Koike and Chaki, 2014). Downstream molecular changes associated with these effects include engagement of the mTOR pathway and modulation of BDNF/TrkB signaling, both of which are implicated in synaptic plasticity and have been linked to antidepressant-like outcomes in animal models (Dwyer et al., 2012; Koike et al., 2013). These processes are proposed to support structural and functional plasticity, such as increased dendritic spine density and synaptogenesis, which may contribute to normalization of dysfunctional neural circuits associated with depressive phenotypes.

Recent preclinical studies employing selective negative allosteric modulators of mGluR2 or mGluR3 have demonstrated that modulation of either receptor subtype can rapidly increase excitatory activity in the medial prefrontal cortex (mPFC) and produce antidepressant-like behavioral effects (Joffe et al., 2020). Specifically, the mGluR2 negative allosteric modulator VU6001966 was reported to preferentially enhance glutamate release along thalamo-cortical pathways, whereas the mGluR3 negative allosteric modulator VU0650786 appeared to influence synaptic plasticity through partially distinct circuit-level mechanisms. These findings suggest that mGluR2 and mGluR3 may engage different molecular and network processes while converging on similar antidepressant-like outcomes. Consistent with this view, enhanced excitatory drive within the mPFC, together with subsequent structural and functional plasticity, has been proposed as a shared mechanism underlying the rapid behavioral effects of mGluR2/3 negative allosteric modulators (Potter et al., 2020).

Despite encouraging preclinical findings, translation to clinical efficacy has proven challenging. Umbricht et al. (2020) conducted a randomized, double-blind, placebo-controlled study examining adjunctive treatment with the mGluR2/3 negative allosteric modulator decoglurant in patients with treatment-resistant depression who had not responded adequately to SSRI or SNRI therapy. Over a 6-week treatment period, decoglurant did not demonstrate statistically significant antidepressant efficacy compared with placebo. However, the compound was generally well tolerated, suggesting that limitations in efficacy may relate to factors such as patient heterogeneity, dosing strategies, or insufficient target engagement rather than safety concerns alone.

A comprehensive review by Jiang et al. (2023) summarized evidence indicating that non-selective mGluR2/3 antagonists and negative allosteric modulators, including MGS0039 and LY341495, produce relatively rapid and sustained antidepressant-like effects in preclinical models when compared with conventional monoaminergic antidepressants. These compounds appear to act through multiple interacting mechanisms, including enhancement of AMPA receptor–dependent synaptic plasticity and indirect modulation of monoaminergic systems. On the basis of these findings, mGluR2/3 antagonists and negative allosteric modulators have been proposed to share certain mechanistic features with ketamine while potentially exhibiting a more favorable side-effect profile, including reduced risk of dissociation or abuse liability (Witkin, 2020; Chaki and Watanabe, 2023). Taken together, pharmacological targeting of mGluR2/3 represents an emerging and actively investigated approach to antidepressant development that is mechanistically distinct from classical monoamine-based therapies and may complement existing glutamatergic strategies.

### 5.10 Psilocybin

In recent years, a growing body of evidence has suggested that certain hallucinogenic compounds may produce rapid and sustained antidepressant effects. Many of these substances, particularly those derived from plants or fungi, have been used for centuries in religious, ceremonial, or spiritual contexts across diverse cultures, largely due to their ability to induce altered states of consciousness and profound subjective experiences. Compounds that elicit perceptual distortions and changes in cognition are collectively referred to as psychedelics. Among these, psilocybin—the principal psychoactive component of so-called “magic mushrooms”—is one of the earliest documented examples.

Archaeological evidence suggests that the use of psilocybin-containing mushrooms may date back several millennia. Rock art discovered at Tassili in the Algerian Sahara, dated to approximately 3500 BCE, depicts figures interpreted as shamans holding mushrooms while dancing, which has been proposed to indicate ritualistic use of psychoactive fungi (Matsushima et al., 2009). In Japan, early literary sources such as the Konjaku Monogatari-shū (circa 1000 CE) contain descriptions suggestive of psychoactive mushroom use, indicating an early awareness of their psychotropic properties.

According to Matsushima et al. (2009), psilocybin was introduced to Europe following the Spanish conquest of the Aztec Empire. Documentation by Fray Bernardino de Sahagún described the ritual consumption of teonanácatl (“divine flesh”), noting its intoxicating and hallucinogenic effects, while Francisco Hernández de Toledo recorded several psychoactive mushrooms regarded as sacred by indigenous populations. Despite Hernández transporting specimens to Spain, their psychoactivity was reportedly lost during transit, and scientific interest in these mushrooms diminished. This knowledge was largely absent from Western discourse until the 20th century, when Richard Schultes reported ritual use of psychoactive mushrooms among the Mazatec people in Mexico in 1938.

Modern scientific interest in psilocybin increased markedly after the publication of the 1957 Life magazine article “Seeking the Magic Mushroom,” which described Gordon Wasson’s participation in Mazatec rituals. This account influenced Timothy Leary, who subsequently initiated the Harvard Psilocybin Project. Although these early investigations contributed to public awareness of psychedelics, methodological limitations and ethical controversies ultimately curtailed academic research in this area, culminating in Leary’s dismissal from Harvard in 1963 (Meyer and Quenzer, 2018).

In recent decades, psilocybin has re-emerged as a candidate for antidepressant treatment. Clinical studies have reported that limited administrations of psilocybin, typically combined with structured psychological support, can produce rapid reductions in depressive symptoms that persist for weeks to months (Carhart-Harris et al., 2018; 2018). Subsequent randomized clinical trials have further supported the presence of rapid-onset and relatively sustained antidepressant effects (Davis et al., 2021; von Rotz et al., 2023). However, these findings are generally based on small sample sizes and controlled settings, and their generalizability remains an active area of investigation.

Several non-mutually exclusive hypotheses have been proposed to account for the rapid and durable antidepressant effects observed following psilocybin administration. One framework emphasizes the role of subjective or “transformative” experiences, suggesting that psilocybin-induced mystical-type experiences may alter cognitive and emotional perspectives in ways that correlate with clinical improvement (Carhart-Harris et al., 2018). A second hypothesis posits that psilocybin enhances psychological flexibility or suggestibility, thereby augmenting the efficacy of concurrent psychotherapy. In parallel, neurobiological mechanisms have been proposed, including activation of serotonin 5-HT_2A_ receptors and downstream changes in synaptic plasticity, potentially involving AMPA receptor-mediated signaling and BDNF-related pathways.

The rapid expansion of research in this field, often referred to as the “Psychedelic Renaissance,” has resulted in a substantial increase in both preclinical and clinical studies worldwide (Tullis, 2021). Despite this progress, the precise molecular and circuit-level mechanisms underlying the antidepressant effects of psychedelics remain incompletely understood.

Neuropharmacological studies have primarily focused on the involvement of 5-HT_2A_ receptors and AMPA receptors. Takaba et al. (2024) examined the effects of several 5-HT_2A_ receptor agonists, including psilocin, DOI, and TCB-2, and demonstrated that blockade of 5-HT_2A_ receptors attenuated antidepressant-like responses, suggesting that 5-HT_2A_ receptor activation may contribute to these effects. In contrast, other findings indicate that psilocybin’s antidepressant actions may occur independently of canonical 5-HT_2A_ receptor signaling (Sekssaoui et al., 2024), raising the possibility that hallucinogenic and antidepressant effects may be mediated by partially dissociable mechanisms. Supporting this notion, Pogorelov et al. (2023) reported that lisuride, a G protein–biased 5-HT_2A_ receptor agonist with minimal hallucinogenic properties, produces antidepressant effects, suggesting that biased signaling at the 5-HT_2A_ receptor may allow separation of therapeutic and hallucinogenic outcomes. Moreover, Muir et al. (2024) demonstrated that administration of DOI to the prefrontal cortex activated neuronal populations that only partially overlapped with cells expressing 5-HT_2A_ receptors. Reactivation of these DOI-responsive neuronal ensembles induced anxiolytic effects without hallucinatory-like behaviors, implying that downstream network-level mechanisms may play a critical role in therapeutic responses.

AMPA receptors have also been implicated in psychedelic-related effects. Berthoux et al. (2018) showed that DOI exposure induced long-term depression of AMPA receptor-mediated synaptic responses in layer V cortical neurons, an effect absent in 5-HT_2A_ receptor knockout mice. Additionally, Zhang & Marek (2008) reported that DOI-induced head-twitch responses were modulated by AMPA receptor agonists, suggesting interactions between serotonergic and glutamatergic signaling in hallucinogen-associated behaviors. Nevertheless, direct evidence linking AMPA receptor modulation to antidepressant efficacy remains limited.

The contribution of 5-HT_2A_ receptor activation and AMPA receptor-mediated plasticity to the antidepressant effects of psilocybin remains an area of active debate. While psilocybin has demonstrated the capacity to induce prolonged antidepressant effects following relatively few administrations, further preclinical and clinical research is required to clarify its mechanisms of action, optimize treatment protocols, and assess long-term safety. Elucidating these mechanisms may facilitate the development of novel psychiatric treatments that differ fundamentally from conventional monoaminergic antidepressants.

## 6 Neural circuits of depression

### 6.1 Serotonergic neurotransmission and information coding in the dorsal raphe nucleus

The serotonergic neurotransmission system of the dorsal raphe nucleus, the primary source of forebrain serotonin, plays a central role in current efforts to understand the neural circuitry underlying depression. According to the classical monoamine hypothesis, depressive states are associated with reduced levels of monoamines, including serotonin, and antidepressant efficacy is achieved by increasing monoaminergic transmission through pharmacological agents such as SSRIs and SNRIs. In contrast to this framework, stress exposure has been reported to induce hyperactivity of the dorsal raphe nucleus (Amat et al., 2005), highlighting an apparent discrepancy between dorsal raphe nucleus activity and serotonergic neurotransmission.

To reconcile this issue, it is essential to consider the diversity and functional properties of serotonin receptors expressed within the dorsal raphe nucleus. At least 14 serotonin receptor subtypes have been identified, including the 5-HT_1_ family (5-HT_1A_, 5-HT_1B_, 5-HT_1D_, 5-HT_1E_, 5-HT_1F_), 5-HT2 family (5-HT_2A_, 5-HT_2B_, 5-HT_2C_), 5-HT_3_, 5-HT_4_, 5-HT_5A_, 5-HT_5B_ (not expressed in humans), 5-HT_6_, and 5-HT_7_ receptors (Meyer et al., 2022). With the exception of the ionotropic 5-HT_3_ receptor, all serotonin receptors are G protein–coupled receptors. Notably, receptors belonging to the 5-HT_1_ family are coupled to G_i/o_ proteins and mediate inhibitory signaling (Polter and Li, 2010). Within the dorsal raphe nucleus, 5-HT_1A_ receptors are predominantly expressed on serotonergic neurons themselves (Huang et al., 2019). These inhibitory receptors function as autoreceptors, such that serotonin binding to 5-HT_1A_ receptors suppresses serotonergic neuronal firing and neurotransmitter release. Accordingly, stress-induced increases in dorsal raphe nucleus activity and extracellular serotonin levels are thought to activate these autoreceptors, resulting in a net inhibition of serotonergic neurotransmission.

Functionally, dorsal raphe nucleus serotonergic neurons have been implicated in the encoding of reward-related information. These neurons have been shown to increase firing in response to reward prediction and acquisition (Hayashi et al., 2015; Inaba et al., 2013; Li et al., 2016; Nakamura et al., 2008), and their activation has been associated with prolonged waiting for delayed rewards (Miyazaki et al., 2014) as well as enhanced reward-seeking behavior (Fonseca et al., 2015; Lottem et al., 2018). Importantly, however, dorsal raphe nucleus neurons do not exclusively encode reward signals. Instead, they respond to both rewarding and aversive stimuli, often across distinct temporal scales, thereby supporting the encoding of both positive and negative motivational values (Hayashi et al., 2015; Cohen et al., 2015).

In addition to serotonergic neurons, the dorsal raphe nucleus contains dopaminergic, glutamatergic, and GABAergic neuronal populations. These cell types partially overlap and interact with one another, modulating local circuit activity and contributing to the integration of complex emotional and motivational information within the dorsal raphe nucleus (Luo et al., 2015). In the following sections, neural circuits centered on the dorsal raphe nucleus that are relevant to depressive states are discussed in further detail.

### 6.2 The medial prefrontal cortex–dorsal raphe nucleus circuit

Not all forms of stress uniformly induce depressive-like behaviors. A critical distinction exists between uncontrollable stress, which reliably produces learned helplessness and depression-like phenotypes, and controllable stress, which typically results in minimal behavioral impairment (Overmier and Seligman, 1967; Seligman, 1972). To address the neural mechanisms underlying this distinction, Amat et al. (2005) investigated which brain regions evaluate stress controllability and how this information modulates serotonergic activity in the dorsal raphe nucleus.

In their study, rats were assigned to three experimental conditions: 1) a controllable stress group, in which footshock could be terminated by wheel running; 2) an uncontrollable stress group, which received identical footshock exposure without behavioral control; and 3) a home-cage control group with no stress exposure. The authors combined pharmacological inactivation of the ventral medial prefrontal cortex (vmPFC) using muscimol with microdialysis measurements of extracellular serotonin levels in the dorsal raphe nucleus, as well as behavioral assessments including fear conditioning and shuttle-box avoidance learning.

Prior anatomical studies had demonstrated that cortical projections to GABAergic neurons in the dorsal raphe nucleus originate predominantly from the vmPFC, and that glutamatergic inputs from the vmPFC may regulate serotonergic neuron activity indirectly via local GABAergic interneurons (Peyron et al., 1998; Vertes, 2004; Jankowski and Sesack, 2004). Consistent with this circuitry, Amat et al. (2005) found that temporary inactivation of the vmPFC abolished the characteristic suppression of dorsal raphe nucleus serotonergic activity normally observed during controllable stress. Specifically, vmPFC inactivation led to increases in c-Fos expression in dorsal raphe nucleus serotonergic neurons and elevations in extracellular serotonin levels during controllable stress to a degree comparable to that observed under uncontrollable stress conditions.

In contrast, vmPFC inactivation had little effect under uncontrollable stress, a condition already associated with robust activation of dorsal raphe nucleus serotonergic neurons. These neurochemical findings were paralleled by behavioral outcomes. When assessed 24 h after stress exposure, rats exposed to controllable stress typically exhibited minimal impairment in fear conditioning and avoidance learning. However, rats in the controllable stress group that underwent vmPFC inactivation displayed enhanced fear conditioning and marked deficits in avoidance behavior, resembling the behavioral profile of rats exposed to uncontrollable stress. Thus, despite experiencing stress that was behaviorally controllable, these animals expressed learned helplessness–like phenotypes.

These findings indicate that while dorsal raphe nucleus serotonergic neurons are activated by aversive stressors *per se*, the vmPFC exerts a top-down inhibitory influence on dorsal raphe nucleus activity when stress is controllable. This regulatory mechanism appears to prevent excessive serotonergic activation and the subsequent development of learned helplessness–like behaviors. The study provides strong evidence that hyperactivation of dorsal raphe nucleus serotonergic neurons contributes to stress-induced behavioral impairments, and that vmPFC-mediated top-down control is a critical component of this process. More broadly, this circuit-level framework suggests that vulnerability to depression-related behaviors is shaped not solely by stress exposure itself, but by neural representations of stress controllability.

### 6.3 Circuits of the lateral habenula

In recent years, accumulating evidence has highlighted the lateral habenula as a key structure in the pathophysiology of depression and depression-like states, alongside the medial prefrontal cortex–dorsal raphe nucleus circuit. The habenula is an evolutionarily conserved brain region present across vertebrate species from fish to mammals, with the medial habenula projecting almost exclusively to the interpeduncular nucleus (Aizawa et al., 2011). In contrast, the lateral habenula receives inputs from limbic and basal ganglia structures and sends outputs primarily to monoaminergic nuclei, including dopaminergic and serotonergic systems (Herkenham and Nauta, 1979). Notably, glutamatergic neurons in the lateral habenula have been shown to preferentially innervate non-serotonergic neurons within the dorsal raphe nucleus that project to the ventral tegmental area (Takahashi et al., 2022).

Consistent with this anatomical organization, the lateral habenula has been proposed to function as a central hub regulating a wide range of behavioral, emotional, cognitive, and stress-related processes, including reward prediction, aversion, motivation, and value-based decision-making (Fortin et al., 2025; Hikosaka, 2010; Hu et al., 2020). Across multiple models of depression—such as chronic stress paradigms, pharmacological treatments, and drug withdrawal—enhanced activity of lateral habenula neurons has been consistently reported (Caldecott-Hazard et al., 1988). Moreover, experimental activation of the lateral habenula has been shown to induce depression-like phenotypes, including anhedonia, reduced social interaction, and other behavioral alterations relevant to depressive states (Chen et al., 2024; Liu et al., 2020; Lecca et al., 2016; Cui et al., 2018; Seo et al., 2018).

Recent studies have also begun to elucidate the molecular and cellular mechanisms underlying this hyperactivity. Reported changes include internalization of GABA_B_ receptors and GIRK channels, leading to a reduction in GABA_B_ receptor–mediated inhibitory currents (Lecca et al., 2016), upregulation of the astrocytic potassium channel Kir4.1 (Cui et al., 2018), and increased expression of the multifunctional protein p11 (Seo et al., 2018). These findings suggest that stress and aversive experiences may induce molecular alterations that disrupt homeostatic regulation within the lateral habenula, thereby contributing to depression-like behavioral outcomes.

Importantly, dysfunction of the lateral habenula offers a perspective on depression that is distinct from circuit models centered on stress controllability, such as the vmPFC–dorsal raphe nucleus pathway. The lateral habenula plays a fundamental role in encoding primary value-related signals—such as reward prediction, aversive value, and arousal level—via its projections to dopaminergic and serotonergic systems (Matsumoto and Hikosaka, 2007; Matsumoto and Hikosaka, 2009). As a result, aberrant lateral habenula function may capture depressive phenotypes driven by alterations in lower-order emotional processing, without necessarily requiring higher-order cognitive appraisal of stress. This framework provides an alternative disease concept that complements cognition-dependent models of depression and broadens the understanding of its neurobiological underpinnings.

### 6.4 Circuit of the median raphe nucleus

To date, the dorsal raphe nucleus, the primary source of serotonergic projections in the brain, has been positioned as a central node in neural circuit models of depression, particularly with respect to stress responses, emotional regulation, and the expression of depressive-like behaviors (Cools et al., 2008; Lowry et al., 2008). However, accumulating evidence has begun to reveal that, in addition to the dorsal raphe nucleus, the median raphe nucleus plays a functionally distinct role in value-based processing of reward and aversion (Ohmura and Nagayasu, 2025).

In a recent study, we demonstrated for the first time in an animal model that selective manipulation of the dorsal raphe nucleus and median raphe nucleus exerts opposing effects on reward- and aversion-related behaviors (Kawai et al., 2022). Specifically, activation of serotonergic neurons in the dorsal raphe nucleus produced reward-related effects, whereas manipulation of serotonergic neurons in the median raphe nucleus induced aversive effects. These findings indicate that the serotonergic system, which has often been treated as a functionally homogeneous neuromodulatory system, is in fact organized into nucleus-specific functional subsystems with distinct and even opposing roles.

The functional dissociation of reward and aversion processing between the dorsal raphe nucleus and median raphe nucleus is likely to be closely linked to the medial prefrontal cortex–dorsal raphe nucleus circuit and the lateral habenula circuit discussed above. These observations suggest that raphe nuclei–specific serotonergic circuits contribute differentially to affective valuation and may play distinct roles in the pathophysiology of depression.

### 6.5 Antidepressants and neural circuits

Selective serotonin reuptake inhibitors (SSRIs) represent the most widely prescribed class of antidepressants. However, they are associated with a well-documented clinical paradox: particularly in younger patients, SSRI treatment has been reported to increase the risk of suicidal ideation during the early phase of treatment (Stone et al., 2009). Although the mechanisms underlying this dual effect have long remained unclear, recent insights into the functional asymmetry between the dorsal raphe nucleus and the median raphe nucleus provide a novel interpretative framework.

From this circuit-based perspective, SSRI-induced elevations in extracellular serotonin do not selectively activate “antidepressant” circuits alone. Rather, increased serotonin levels may simultaneously engage both dorsal raphe nucleus-centered circuits involved in reward and positive affect and median raphe nucleus-centered circuits associated with aversion, anxiety, and avoidance. During the initial phase of treatment, preferential or relatively stronger activation of median raphe nucleus-related aversive and anxiety-related circuits may lead to heightened anxiety and exacerbation of suicidal ideation. In contrast, with continued treatment, progressive reorganization and normalization of reward- and emotion-related circuits—including those centered on the dorsal raphe nucleus—may occur, ultimately giving rise to clinically observable antidepressant effects.

Thus, even a single pharmacological action—namely, increased serotonergic tone—may exert divergent and even opposing emotional effects at the circuit level, reflecting the parallel regulation of neural systems encoding distinct affective values.

### 6.6 Depression as a pathological breakdown of neural circuits

Integrating the medial prefrontal cortex–dorsal raphe nucleus circuit, the lateral habenula circuit, and the functional dissociation between the dorsal and median raphe nuclei leads to a reconceptualization of depression. Rather than being viewed solely as a disorder caused by reduced monoamine levels, depression can be more appropriately understood as a disorder of neural circuits—a *circuitopathy*—characterized by an imbalance between reward- and aversion-related circuits.

Recent advances in neural circuit research increasingly support a shift away from defining depression as a state of “monoamine deficiency” toward framing it as a pathological disruption of the balance between circuits mediating reward and aversion. In particular, the discovery of functional dissociation between the dorsal raphe nucleus and median raphe nucleus provides a unifying framework that may account for both the therapeutic efficacy and adverse effects of SSRIs. This circuit-level perspective offers critical insights that may inform the development of next-generation antidepressants and serve as a foundation for personalized approaches to psychiatric treatment.

## 7 Animal models of depression

### 7.1 Predictive validity, face validity, and construct validity

Extensive preclinical research has been conducted to elucidate the neurobiological mechanisms underlying depression and to facilitate the development of novel therapeutic strategies. Within this context, animal models of depression have played an important role in advancing mechanistic understanding and preclinical drug discovery, while also presenting inherent translational limitations. The validity of such models is commonly evaluated along three conceptual dimensions: predictive validity, face validity, and construct validity. Willner (1984) proposed this framework as a means of systematically assessing the relevance of animal models to clinical depression. Predictive validity refers to the extent to which a model is able to predict the therapeutic efficacy of antidepressant treatments and whether the behavioral or physiological responses observed in the model correspond to clinical outcomes. Face validity describes the degree to which behavioral phenotypes expressed by the model resemble core symptoms observed in patients with depression. Construct validity reflects the extent to which the etiological factors, precipitating conditions, or underlying neurobiological alterations in the model correspond to those implicated in the pathophysiology of clinical depression.

### 7.2 Learned helplessness models

Stress-induced animal models have been widely used to investigate the neurobiological mechanisms underlying depression and to explore potential therapeutic strategies. These models aim to induce depression-relevant behavioral and physiological phenotypes, thereby emphasizing face validity through the reproduction of features commonly observed in human depressive states.

The learned helplessness model was originally proposed by Seligman and Maier (1967). Overmier and Seligman (1967) demonstrated that dogs exposed to inescapable foot shocks subsequently failed to perform avoidance responses even when escape was later made possible. They showed that this deficit in avoidance behavior resulted from the animals learning that they could not control the aversive stimulus through their own actions, and this state was defined as “learned helplessness” (Seligman and Maier, 1967). Conceptually, this model has been regarded as an experimental analogue of depression-related psychological features, such as helplessness and hopelessness, rather than a direct representation of subjective cognitive states (Seligman, 1972).

At the neural level, accumulating evidence indicates that the long-term behavioral consequences of uncontrollable stress critically involve the dorsal raphe nucleus. Maier and Watkins (2005) reported that exposure to uncontrollable stress robustly activates serotonergic neurons within the dorsal raphe nucleus. Pharmacological studies further demonstrated that inhibition of dorsal raphe nucleus activity can prevent the development of learned helplessness–like behaviors, whereas artificial activation of the dorsal raphe nucleus is sufficient to induce behavioral phenotypes resembling those produced by uncontrollable stress. These findings suggest that dorsal raphe nucleus-dependent serotonergic signaling plays an important role in mediating the behavioral effects of uncontrollable stress.

In addition to behavioral alterations, learned helplessness is accompanied by changes in neurotrophic factors and elevations in corticosterone levels, reflecting stress-related neuroendocrine dysregulation that overlaps with biological features frequently reported in depression (Planchez et al., 2019). While these physiological changes are not specific to depression, their presence supports the relevance of this model for studying stress-related mechanisms implicated in depressive pathology.

The learned helplessness model captures key behavioral and neurobiological consequences of uncontrollable stress and provides partial support for construct validity by linking defined environmental stressors to reproducible neural and endocrine alterations. At the same time, the model’s limitations highlight the importance of interpreting its phenotypes as stress-related depressive analogues rather than direct equivalents of clinical depression.

### 7.3 Social defeat model

The social defeat model is a widely used stress-based procedure in which an experimental mouse is repeatedly exposed to an aggressive conspecific, typically within the aggressor’s home cage for a limited period each day, followed by sensory contact through a partition. In a commonly used protocol, this procedure is repeated over approximately 10 consecutive days. Under these conditions, a substantial proportion of mice—often reported to be around two-thirds—develop behavioral alterations such as reduced social interaction, changes in reward-related behavior, and physiological alterations consistent with stress exposure, whereas the remaining animals show relative resistance to these effects (Golden et al., 2011). Although the specific behavioral outcomes can vary depending on experimental conditions, mouse strain, and assessment methods, social avoidance has emerged as a robust and reproducible phenotype used to classify stress-susceptible and stress-resilient individuals.

Importantly, exposure to repeated social defeat induces not only depression-related behavioral changes but also anxiety-like responses and elevations in corticosterone levels across experimental groups, highlighting the overlap between depressive- and anxiety-related phenotypes in this model (Golden et al., 2011). As a result, the social defeat procedure has been particularly informative for investigating individual differences in vulnerability and resilience to stress, rather than modeling a single, unitary depressive syndrome. At the circuit level, resilience to social defeat has been associated with functional adaptations within reward-related neural networks, including the ventral tegmental area, nucleus accumbens, and prefrontal cortex (Nestler and Russo, 2024).

From a pharmacological perspective, the social defeat model exhibits predictive validity in that both rapid-acting and conventional antidepressants can ameliorate specific stress-induced behavioral deficits. For example, administration of ketamine (20 mg/kg) prior to social interaction testing following repeated social defeat has been shown to attenuate social avoidance behavior (Donahue et al., 2014). In addition, chronic treatment with classical antidepressants such as imipramine or fluoxetine over several weeks restores social interaction behavior in susceptible mice (Tsankova et al., 2006). Although the repeated social defeat stress paradigm was originally developed and most extensively validated in male mice, modified versions of this model have also been adapted for use in females, enabling the investigation of stress susceptibility and antidepressant responses across sexes (Takahashi et al., 2017). However, sex-specific differences in aggression, social hierarchy formation, and hormonal influences introduce additional complexity, and methodological variability across female social defeat paradigms can complicate direct comparisons with male data. Consequently, while social defeat stress provides a powerful framework for studying stress-induced behavioral and pharmacological phenotypes, a more nuanced and standardized consideration of sex differences remains an important challenge for the field (Hao et al., 2019).

### 7.4 Unpredictable chronic mild stress model

The unpredictable chronic mild stress model exposes animals to a variety of mild stressors in an unpredictable schedule over an extended period. Examples of stressors include wet bedding, tilted cages, disruptions to the light-dark cycle (e.g., 30-min alternating cycles), transfer to previously occupied cages, shallow water exposure, introduction of predator fur or urine, and playback of predator vocalizations. Stressors are applied for approximately 3 h per day, after which animals are returned to clean cages. When applied for 7–9 weeks, unpredictable chronic mild stress produces behavioral and physiological changes such as reduced sucrose preference, increased immobility in the tail suspension test, elevated corticosterone levels, and increased TNF-α expression (Frisbee et al., 2015). Unpredictable chronic mild stress also induces reductions in hippocampal volume, mirroring findings observed in depressed patients (Luo et al., 2014).

However, unpredictable chronic mild stress is generally effective only in certain strains, such as Sprague-Dawley and Wistar rats, and BALB/cJ mice, whereas strains like DBA/2 and C57BL/6 may show limited effects. Concerns regarding reproducibility have been raised; however, Willner (2017) suggested that laboratories reporting null effects may have applied stress for insufficient durations, and that the model is effective when properly implemented. The unpredictability and diversity of stressors, while enhancing ecological validity, also make experimental control challenging, representing an inherent limitation of the unpredictable chronic mild stress model.

Advances in research have elucidated several biological alterations observed in patients with depression, including central monoamine depletion (particularly serotonin and noradrenaline), systemic inflammation, and dysregulation of the HPA axis. Animal models based on these biological findings are designed according to the principle that inducing similar biological changes in experimental animals can recapitulate aspects of the depressive state observed in humans (Planchez et al., 2019).

### 7.5 Maternal separation and social isolation models of depression

The profound influence of maternal separation and social isolation on social and emotional behavior was first demonstrated experimentally through a series of seminal studies by Harry F. Harlow. In an early investigation, Harlow and Zimmermann (1959) reported that infant rhesus monkeys separated from their mothers preferentially clung to a soft cloth surrogate that provided tactile comfort but no nutrition, rather than to a wire surrogate that supplied milk. When exposed to fear-inducing stimuli, the animals consistently sought refuge with the cloth surrogate. These findings indicated that mother–infant attachment is not solely driven by the satisfaction of physiological needs, but is strongly motivated by emotional and social factors. This work subsequently provided an important conceptual foundation for later studies examining the effects of social isolation on emotional development, depression-like behaviors, and models of psychopathology.

Building on this framework, McKinney and Harlow (1972) demonstrated that juvenile rhesus monkeys subjected to social isolation for 10 weeks exhibited increased clinging behavior—considered an index of anxiety—as well as reduced overall activity levels. These findings suggested that social isolation paradigms could be useful for modeling affective disturbances. Early rodent studies further showed that social isolation housing enhanced adrenocortical function and increased corticosterone levels, a physiological marker of stress (Hatch et al., 1965). Consequently, social isolation was initially studied primarily as a model of anxiety-like behavior (Parker and Morinan, 1986; Wright et al., 1991).

The use of maternal separation and social isolation as models of depression-like behavior became more established after 2000. Subsequent studies reported increased immobility in the forced swim test (Martin and Brown, 2010; Gills and Polston, 2017), reduced sucrose preference indicative of anhedonia (Gills and Polston, 2017), and heightened anxiety-like behavior (Hermes et al., 2011). These findings suggest that these manipulations can reliably induce abnormalities in emotional behavior. However, numerous studies have also reported that maternal separation and social isolation can elicit behavioral phenotypes relevant to schizophrenia-like states (Geyer et al., 1993; Varty et al., 2006; Wilkinson et al., 1994). Thus, the behavioral outcomes observed in these models do not consistently map onto depressive symptomatology alone.

In addition, the physiological alterations reported in these paradigms lack consistency across studies. While increases in corticosterone levels have been observed (Hatch et al., 1965; Martin and Brown, 2010), other reports describe elevated dopamine levels (Han et al., 2011) or reduced expression of the AMPA receptor subunit GluR1 (Hermes et al., 2011). Such variability suggests that maternal separation and social isolation induce broad neurobiological changes rather than a uniform depression-specific phenotype.

In summary, substantial evidence supports the notion that maternal separation and social isolation profoundly disrupt emotional and social behaviors. Nevertheless, the resulting phenotypes are heterogeneous and span multiple psychiatric domains. Consequently, while these paradigms are valuable for studying the effects of early-life and social stress, their face validity and construct validity as specific models of depression are limited.

### 7.6 Olfactory bulbectomy model

The olfactory bulbectomy model, generated by bilateral removal of the olfactory bulbs, has been widely used since the 1980s as an experimental model for studying affective disorders, particularly depression (Song and Leonard, 2005). Olfactory bulbectomy induces extensive and reproducible neurobiological alterations, including reduced signal intensity in the cerebral cortex, hippocampus, caudate nucleus, and amygdala, as well as enlargement of the lateral and third ventricles (Wrynn et al., 2000). In addition, olfactory bulbectomy has been shown to reduce hippocampal neurogenesis (Morales-Medina et al., 2017). Some of these changes partially overlap with clinical observations in patients with depression, such as associations between ventricular enlargement and cognitive impairment (Kellner et al., 1986) and reductions in hippocampal volume (Videbech and Ravnkilde, 2004). Moreover, olfactory bulbectomy produces marked alterations in monoaminergic, immune, and endocrine systems (Song and Leonard, 2005), supporting its utility for investigating neurobiological processes relevant to affective pathology and suggesting a degree of construct validity.

However, the behavioral phenotypes observed following olfactory bulbectomy warrant careful interpretation. One of the most consistently reported features of this model is increased locomotor activity and impaired habituation in novel environments (Hellweg et al., 2007; Farina de Almeida et al., 2017; Zueger et al., 2005). In addition, studies using fear conditioning paradigms have demonstrated attenuated fear responses to cues predicting aversive stimuli (Primeaux and Holmes, 2000; King and Cairncross, 1974). These behavioral characteristics differ from those commonly observed in many stress-based depression models, which typically exhibit reduced activity and increased behavioral inhibition.

While heightened exploratory activity could be interpreted as reflecting anxiety-related or disinhibitory states, olfactory bulbectomy animals also show reduced fear responses and impairments in spatial learning in tasks such as the Morris water maze (Zhao et al., 2025). These findings suggest that the behavioral consequences of olfactory bulbectomy may reflect broader disturbances in cognitive and emotional regulation rather than selectively modeling core depressive-like symptoms. In line with this interpretation, recent studies have proposed that olfactory bulbectomy captures certain pathological features relevant to neurodegenerative conditions, including Alzheimer’s disease, particularly with respect to cognitive dysfunction (Bobkova et al., 2025; Zhao et al., 2025).

In summary, although olfactory bulbectomy produces robust neurobiological changes that overlap with some features observed in depression, the associated behavioral phenotypes are heterogeneous and not specific to depressive symptomatology. Therefore, while the olfactory bulbectomy model remains valuable for studying the consequences of widespread neural disruption and cognitive impairment, its face validity as a model of depression should be regarded as limited and highly context-dependent.

### 7.7 Reserpine-induced model

The reserpine-induced model is based on administration of reserpine, a vesicular monoamine transporter 2 (VMAT2) inhibitor that produces a marked depletion of central monoamines. Historically, reserpine was introduced as an antihypertensive agent, and early clinical observations suggested an association between its use and the emergence of depressive symptoms, providing influential support for the monoamine hypothesis of depression (Freis, 1954). In preclinical studies, repeated reserpine administration has been reported to induce a range of behavioral and physiological changes, including weight loss, reduced locomotor activity (de Freitas et al., 2016; Park et al., 2018), anxiety-like behaviors, and anhedonia (Telega et al., 2024). These outcomes have often been interpreted as reflecting depression-related behavioral domains. However, several of these measures—particularly hypolocomotion and weight loss—are difficult to dissociate from non-specific effects such as generalized motor suppression, sickness behavior, or systemic toxicity.

Importantly, the construct validity of the reserpine model as a model of depression has been increasingly questioned. A recent meta-analysis and systematic re-evaluation of clinical and experimental data found little consistent evidence that reserpine reliably induces depressive symptoms in humans, challenging the historical assumption that monoamine depletion *per se* is sufficient to cause depression (Strawbridge et al., 2023). These findings substantially weaken the translational rationale of the reserpine model and call into question its continued use as a primary experimental model of depression.

Methodological limitations further constrain the utility of this model. There is no consensus regarding optimal dosing regimens or administration schedules, contributing to substantial variability across studies (Telega et al., 2024). Moreover, depending on dose and duration, reserpine treatment has been associated with increased mortality and systemic adverse effects, limiting experimental feasibility and interpretability.

Consistent with these concerns, the predictive validity of the reserpine model is also limited. The ability of monoamine-enhancing antidepressants—including TCAs, SSRIs, SNRIs, and NaSSAs—to reverse reserpine-induced behavioral alterations has been inconsistent across studies, likely reflecting heterogeneity in experimental design, behavioral endpoints, and treatment parameters.

While the reserpine-induced model retains historical significance and may still be useful for probing the consequences of profound monoaminergic depletion, contemporary clinical and meta-analytic evidence indicates that it lacks strong construct validity as a model of depression. Accordingly, its use in depression research should be interpreted with caution and framed within a narrow mechanistic context rather than as a comprehensive model of depressive pathology.

### 7.8 Corticosterone administration model

The corticosterone administration model is widely used to examine depression-relevant behavioral and neuroendocrine alterations by chronically administering corticosterone, the primary glucocorticoid released following activation of the HPA axis in rodents (cortisol in humans). Chronic corticosterone exposure has been reported to induce a range of behavioral changes relevant to depression, including alterations in body weight, increased immobility in the forced swim test (Johnson et al., 2006; Shoji et al., 2024; Sturm et al., 2015), enhanced anxiety-like behavior, reduced social interaction (Shoji et al., 2024), and decreased sucrose solution intake (Shoji et al., 2024; Sturm et al., 2015). These findings suggest that prolonged elevation of glucocorticoids can influence multiple behavioral domains commonly affected in depressive states.

However, important limitations of this model should be considered. The endocrine profile produced by exogenous corticosterone administration does not fully replicate the dynamic regulation of the HPA axis observed following chronic stress exposure. Although corticosterone levels rise acutely after administration, the circadian nadir of corticosterone secretion remains largely intact (Kinlein et al., 2015). This dissociation indicates that the model captures sustained glucocorticoid signaling rather than the full spectrum of stress-induced HPA axis dysregulation.

Accordingly, the corticosterone administration model may be best viewed as a tool for investigating glucocorticoid-driven mechanisms contributing to depression-related behaviors, rather than as a comprehensive model of stress-induced depression. Its utility lies in isolating the effects of prolonged glucocorticoid exposure on emotional and social behaviors, while its face and construct validity should be interpreted in light of these physiological constraints.

### 7.9 Lipopolysaccharide model

The lipopolysaccharide (LPS) administration model induces depression-relevant behavioral alterations by activating peripheral and central inflammatory responses. With increasing recognition of the role of inflammation in the pathophysiology of depression, this model has been widely employed to investigate inflammation-associated mechanisms contributing to depressive symptomatology. Two principal experimental paradigms are commonly used: an acute single-dose paradigm, in which behavioral changes emerge within approximately 24 h after injection, and a chronic low-dose paradigm designed to model sustained inflammatory states.

Following acute LPS administration, robust elevations in proinflammatory cytokine levels have been reported in both peripheral circulation and brain tissue within 24 h (Tonelli et al., 2008). This inflammatory response is accompanied by physiological and behavioral alterations, including reductions in body weight (Banks et al., 2015; Piirsalu et al., 2020) and decreased spontaneous locomotor activity (Tang et al., 2020). In addition, increased immobility in the forced swim test and tail suspension test has been observed (Li et al., 2015; Tastan et al., 2021; Walker et al., 2013; Li et al., 2021), along with reduced sucrose preference, which is often interpreted as an anhedonia-like phenotype (Salazar et al., 2012; Guo et al., 2014).

Importantly, many of the behavioral changes observed shortly after acute LPS exposure overlap with features of sickness behavior, such as reduced activity and weight loss, which arise as adaptive responses to systemic inflammation. Accordingly, interpretation of depression-like behaviors in the acute LPS model requires careful consideration of potential confounding effects related to generalized malaise or reduced motor capacity. Nevertheless, the concurrent emergence of motivational deficits, including decreased sucrose preference, suggests that inflammatory activation can engage neural processes relevant to depressive symptom domains.

Compared with reserpine-induced or chronic corticosterone administration models, the acute LPS paradigm offers the advantage of rapid and reproducible induction of inflammation-associated behavioral alterations. However, a notable limitation of the single-dose model is the transient nature of these effects, with depression-relevant behaviors typically diminishing within approximately 48 h post-injection. This temporal profile indicates that the acute LPS model may be particularly suited for investigating short-term interactions between immune activation and mood-related behaviors, rather than modeling the persistent symptomatology characteristic of major depressive disorder.

### 7.10 Limitations of animal models

By comparing commonly used paradigms in terms of dominant signaling pathways and translational relevance, we aim to clarify how different models capture distinct aspects of depressive pathology. A summary of the advantages and limitations of representative models is provided in Table 1. A comparative overview of stress-induced and biologically based models of depression reveals substantial differences in their validity, advantages, and inherent limitations. Stress-induced models, although often labor-intensive and sometimes involving exposure to aversive stimuli, are widely used because they recapitulate key aspects of how depressive-like states emerge following stressful experiences. In this respect, these models are often considered to possess relatively high face validity, as they reproduce selected behavioral and physiological features associated with depressive episodes in humans. In addition, some stress-induced models exhibit partial construct validity, insofar as they engage neuroendocrine, monoaminergic, and immune-related changes that overlap with those reported in patients with depression. Moreover, depression-like behaviors induced by stress are frequently ameliorated by antidepressant treatments, supporting a degree of predictive validity.

**TABLE 1:** Comparative overview of animal models of depression.

Model category	Representative models	Dominant signaling pathways	Key strengths	Major limitations	Translational relevance
Stress-based	Social defeat, UCMS	HPA axis, monoamines, plasticity	Face validity, resilience	Time, variability	High (stress-related)
Pharmacological	Reserpine	Monoamines	Simplicity, control	Low specificity	Low
Endocrine	Corticosterone	Glucocorticoid receptor	Isolates hormone effects	Lacks dynamics	Moderate
Inflammation -based	LPS	Cytokines, microglia	Immune relevance	Sickness confound	Subtype (inflammation) ‐specific

Nevertheless, important limitations must be acknowledged. In the learned helplessness model, both experimental and control animals are exposed to electric shocks, indicating that nociceptive stimulation alone is insufficient to account for the emergence of depression-like behaviors. Conversely, in the social defeat model, physical injury resulting from aggressive encounters is not always independently controlled, making it difficult to fully dissociate the effects of social stress from those of pain or injury. More broadly, stress-induced models often require substantial technical expertise and prolonged experimental timelines, which can limit their accessibility and reproducibility across laboratories.

Biologically based models, in contrast, aim to induce depression-like phenotypes by directly manipulating physiological processes implicated in depression, thereby emphasizing construct validity. However, their translational relevance is not always straightforward. For example, in the LPS administration model, the doses commonly used in rodents induce robust cytokine elevations and sickness responses that differ markedly from the effects of much lower doses administered in human experimental endotoxemia studies, highlighting translational challenges in comparing acute high-dose rodent inflammation with low-grade immune activation in depressed patients (Lasselin et al., 2020). Similarly, the reserpine-induced model has been increasingly scrutinized, as recent clinical evidence does not consistently support the notion of globally reduced serotonin levels in depression, highlighting limitations of a simplistic interpretation of the serotonin hypothesis (Moncrieff et al., 2023).

Despite these caveats, biologically based models offer clear experimental advantages. The physiological alterations they induce are often well characterized and reproducible, facilitating mechanistic investigations. For instance, pre-administration of compounds sharing ketamine’s mechanism of action has been shown to prevent reserpine-induced depression-like behaviors (Gao et al., 2016), demonstrating the utility of such models for testing specific pathophysiological hypotheses. In addition, their procedural simplicity—often involving only pharmacological manipulation—makes them particularly attractive for high-throughput screening and pharmaceutical research, where experimental speed and standardization are critical.

Importantly, no single animal model fully captures the complexity of depression, reflecting the multifactorial nature of the disorder and the dynamic interplay between biological vulnerability and environmental influences. While genetic and developmental risk factors contribute to susceptibility, depressive disorders rarely manifest independently of experience. Rather, depression typically emerges through experience-dependent learning processes, in which repeated exposure to stress shapes maladaptive behavioral responses, such as passive coping, behavioral withdrawal, and reduced motivation. In this context, Seligman’s concept of learned helplessness remains highly influential, as it explicitly conceptualizes depressive-like behavior as the consequence of learning from uncontrollable or adverse experiences. This framework is consistent with accumulating biological evidence implicating glutamatergic signaling and synaptic plasticity—core mechanisms of learning and memory—as central contributors to the pathophysiology of depression and to the actions of rapid-acting antidepressants. Accordingly, stress-induced models that capture experience-dependent behavioral adaptation continue to provide valuable insights into both the behavioral and neurobiological mechanisms underlying depressive episodes.

Integrating evidence from both experimental models and clinical studies has progressively clarified the biological underpinnings of depression. Future advances may arise from hybrid approaches that combine stress-based and biologically based paradigms. For example, models in which mild inflammatory activation via LPS or monoamine depletion via reserpine is followed by stress exposure may provide a useful experimental framework for investigating how biological vulnerability interacts with environmental stress, thereby offering a more comprehensive representation of depressive pathology.

The translational relevance of depression models depends on the specific biological processes under investigation, the signaling pathways involved, and their temporal dynamics. Consequently, comparative evaluation of stress-induced, pharmacological, endocrine, and inflammation-based paradigms provides a more integrative framework for selecting appropriate experimental models in preclinical research. Future studies should aim to characterize depression-like behaviors and their underlying physiological mechanisms across multiple models, integrating both psychological and biological perspectives.

## 8 Conclusion

Despite decades of research, major depressive disorder remains a complex and heterogeneous condition with no single unifying pathophysiological explanation. Early frameworks centered on monoaminergic dysfunction provided a foundation for the development of widely used antidepressants, yet these treatments are limited by delayed therapeutic onset, incomplete efficacy, and notable adverse effects. The emergence of rapid-acting antidepressants, including ketamine and psychedelic compounds, has shifted the field toward novel mechanisms involving glutamatergic signaling, synaptic plasticity, and neuroimmune interactions, challenging long-held assumptions about the biology of depression.

Parallel advances in preclinical research have demonstrated the value and limitations of diverse animal models. Stress-based paradigms, such as learned helplessness, social defeat, and unpredictable chronic mild stress, capture key environmental contributors to depression and show strong face and predictive validity. Biologically driven models—including reserpine administration, chronic corticosterone exposure, and LPS-induced inflammation—offer mechanistic specificity but vary widely in construct validity relative to human pathology. These models illustrate that no single approach can fully recapitulate the multifaceted nature of depression; instead, their complementary strengths provide a multifactorial framework for understanding disease processes.

Accumulating evidence suggests that depressive states arise through dynamic interactions between genetic vulnerabilities, environmental stressors, neuromodulatory systems, and immune mechanisms. Integrative models that combine stress exposure with defined biological perturbations may therefore offer improved translational relevance, capturing the interplay between environmental experience and neurobiological susceptibility.

Looking forward, a major priority for depression research will be the identification of biologically informed biomarkers capable of linking molecular, circuit, and behavioral phenotypes. Such biomarkers may facilitate patient stratification, enabling the classification of depressive subtypes based on circuit dysfunction, inflammatory status, or plasticity-related signaling rather than symptom-based criteria alone. This approach holds particular promise for predicting treatment responsiveness to rapid-acting versus conventional antidepressants. In parallel, advances in circuit-mapping and cell-type–specific manipulation have highlighted the therapeutic potential of targeting defined neural subcircuits and cellular populations within mood-related networks. Emerging signaling frameworks centered on glutamatergic transmission, synaptic plasticity, and neuroimmune interactions provide mechanistic entry points for the development of next-generation antidepressants that move beyond global neurotransmitter modulation toward more precise and durable interventions.

The integration of refined animal models, translational biomarkers, and circuit- and cell-type–specific therapeutic strategies provides a foundation for advancing toward a systems-level understanding of depression. Such progress will be essential for the development of faster-acting, more effective, and safer treatments capable of addressing the substantial unmet clinical needs of individuals living with depression. However, achieving a truly systems-level understanding of depression will require not only advances in molecular and circuit-level analyses, but also a re-examination of how depression-related behaviors are defined and quantified in preclinical models.

At the same time, while advances in molecular biology, pharmacology, and systems neuroscience have dramatically expanded our ability to interrogate neural circuits and signaling pathways, progress in the behavioral characterization of depression-related phenotypes has lagged behind these advances, remaining comparatively limited. Traditional behavioral readouts in animal models often rely on coarse or endpoint measures, such as immobility duration or avoidance indices, which may insufficiently capture the richness and dynamics of affective states and coping strategies. Recent developments in image-based behavioral analysis, machine learning–assisted pose estimation, and computational modeling now offer powerful tools to quantify behavior with greater temporal, spatial, and conceptual resolution (Datta et al., 2019; Mathis et al., 2018; Weinreb et al., 2024; Wiltschko et al., 2015). These approaches enable the extraction of latent behavioral features and state transitions that are more directly linked to underlying neural and computational processes. Incorporating such quantitative and computational frameworks into stress-based and pharmacological models of depression represents a critical step toward improving translational validity.

By integrating advanced behavioral analytics with circuit-, cellular-, and molecular-level investigations, future translational approaches may more effectively bridge preclinical models and clinical phenotypes. Such integration holds the potential not only to refine animal models of depression but also to accelerate the identification of behaviorally grounded biomarkers and the development of antidepressant therapies with improved precision and efficacy. Ultimately, redefining depression-related behaviors in quantitative and computational terms may be as essential to next-generation antidepressant discovery as advances in molecular targeting and circuit-level intervention.
